# Supplementary material for: Food Pleasure Profiles—An Exploratory Case Study of the Relation between Drivers of Food Pleasure and Lifestyle and Personality Traits in a Danish Consumer Segment
Source: Foods. 2022 Feb 28;11(5):718. doi: 10.3390/foods11050718 (PMC8909624; doi:10.3390/foods11050718)
Supplement: Supplementary file 1 [file foods-11-00718-s001.zip › Supp-3rd revised.pdf]

**Supplementary Material**

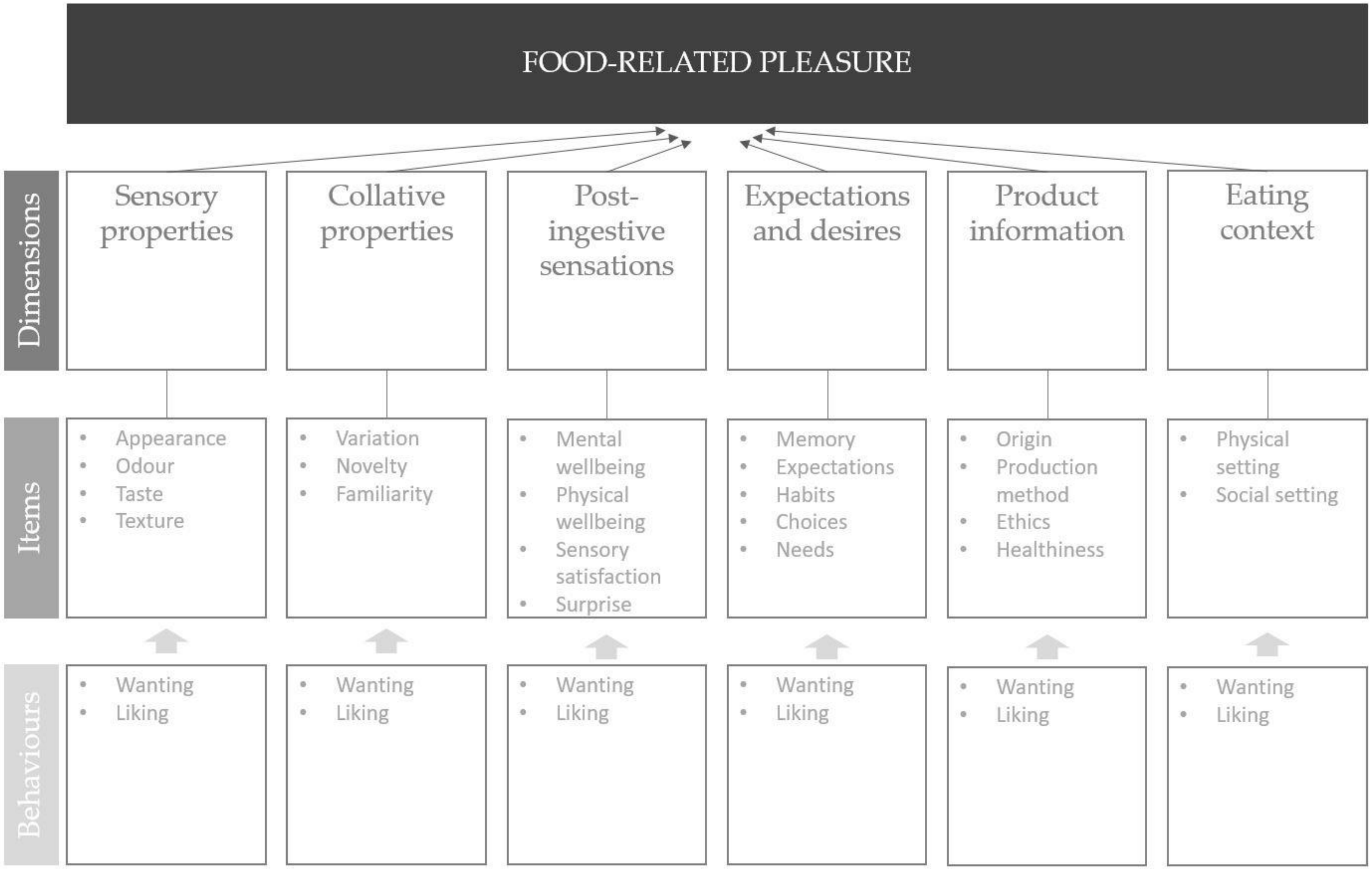


**Figure S1**. Schematic conceptualisation of the key dimensions, items, and behavioural elements involved in the individual food-related pleasure response, allowing a holistic study of quantitative (level of pleasure) and qualitative (drivers of pleasure) aspects of food-related pleasure. The model is kindly borrowed from Andersen, B. V., Chan, R. C. K., & Byrne, D. V. (2021). A Conceptual Framework for Multi-Dimensional Measurements of Food Related Pleasure—The Food Pleasure Scale. Foods, 10(9), 2044. https://doi.org/10.3390/foods10092044

**Table S1**. Response variables with English and Danish phrasings as used in the questionnaires. Data were collected on a 5-point ordinal scale, where “1”=”Not important at all, “2”=”Not important”, “3”=”Neither important nor not important”, “4”=”Important” and “5”=”Extremely important”.

| | English phrasing | Danish Phrasing |
|---|---|---|
| Question for each item | “How important is the following for your experience of food pleasure when eating?” | ”Hvor vigtigt er følgende for din generelle oplevelse af nydelse ved fødevarer, når du spiser?” |
| Item | | |
| Memory | “To experience positive memories of food” | ”At opleve positive minder om mad” |
| Need | “To experience satisfying a need” | ”At opleve at få tilfredsstillet et behov” |
| Choice | “To experience having choices in the eating situation” | ”At opleve at have valgmuligheder i spisesituationen” |
| Habit | “To be able to maintain a habit” | ”At kunne bibeholde en vane” |
| Physical eating setting | “To experience positive physical surroundings” | ”At opleve en positiv fysisk ramme” |
| Eating w. others | “To experience eating with others” | ”At opleve at spise sammen med andre” |
| Eating alone | “To experience eating food when I am alone” | ”At opleve at spise mad, når jeg er alene” |
| Variation | “To experience food variation” | “At opleve madvariation” |
| Familiarity | “To experience familiar food” | “At opleve bekendt mad” |
| Novelty | “To experience new (unknown) food” | ”At opleve ny (ukendt) mad” |
| Food Appearance | “The appearance of the food” | “Madens udseende” |
| Food Odour | “The odour of the food” | “Madens duft” |
| Food Taste | “The taste / flavour of the food” | “Madens smag” |
| Food Texture | “The texture of the food” | “Madens tekstur” |
| Pleased senses | “To experience a satisfaction of the sense of sight, smell, taste and touch” | ”At opleve en tilfredsstillelse af syns-, lugte-, smags- og fornemmelses-sansen” |
| Physical sensation | “To experience a positive physical sensation in the body after eating” | ”At opleve en positiv fysisk fornemmelse i kroppen efter spisning” |
| Mental sensation | “To experience a positive mental feeling / sensation after eating” | ”At opleve en positiv mental fornemmelse / følelse efter spisning” |
| Surprise | “To experience positive surprises from the meal” | ”At opleve positive overraskelser ved maden” |
| Atmosphere | “To experience a positive atmosphere” | ”At opleve en positiv stemning / atmosfære” |

**Table S2.** Overview of distributions of all variables by the five segments and the 'Tie on first' subgroup as well as results of Chi $X^2$ / Kruskal-Wallis tests.

| | Total | Sensory Pleasure Seekers | Exploratory Pleasure Seekers | Contextual Pleasure Seekers | Confirming Pleasure Seekers | Internal Pleasure Seekers | Tie on first | Chi $X^2$. df. | p-value |
|---|---|---|---|---|---|---|---|---|---|
| | $N_{total}$=355 | N=176 (50%) | N=46 (13%) | N=60 (17%) | N=18 (5%) | N=121 (34%) | N=46 (13%) | | |
| **Socio-demographic variables** | | | | | | | | | |
| **Gender, Female/Male** | 284/71 (80%/20%) | 142/34 (81%/19%) | 35/11 (76%/24%) | 47/13 (78%/22%) | 11/7 (61%/39%) | 95/26 (79%/21%) | 33/13 (72%/28%) | 3.18, df = 5 | 0.673 |
| **Age, mean (±SD)** | 33.34 ±13.07 | 34.71 (12.85) | 29.03 (9.82) | 35.16 (13.38) | 40.60 (17.50) | 32.71 (13.71) | 35.04 (12.90) | 50.04, df = 47 | 0.354* |
| **Educational level** | | | | | | | | 48.28, df = 35 | 0.067 |
| Primary School | 14 (4%) | 7 (4%) | 3 (7%) | 1 (2%) | 0 (0%) | 5 (4%) | 1 (2%) | | |
| High School | 115 (32%) | 47 (27%) | 20 (43%) | 16 (27%) | 7 (39%) | 48 (40%) | 12 (26%) | | |
| Vocational Education | 30 (8%) | 20 (11%) | 2 (4%) | 1 (2%) | 3 (17%) | 8 (7%) | 4 (9%) | | |
| Short Higher Education | 30 (8%) | 15 (9%) | 3 (7%) | 6 (10%) | 2 (11%) | 12 (10%) | 7 (15%) | | |
| Medium Higher Education | 82 (23%) | 47 (27%) | 7 (15%) | 16 (27%) | 3 (17%) | 19 (16%) | 7 (15%) | | |
| Long Higher Education | 74 (21%) | 37 (21%) | 10 (22%) | 17 (28%) | 3 (17%) | 25 (21%) | 14 (30%) | | |
| PhD | 9 (3%) | 3 (2%) | 0 (0%) | 3 (5%) | 0 (0%) | 4 (3%) | 1 (2%) | | |
| **Socioeconomic Status** | | | | | | | | 59.81, df = 25 | <0.001 |
| Student | 130 (37%) | 53 (30%) | 18 (49%) | 19 (32%) | 4 (22%) | 52 (43%) | 12 (26%) | | |
| Unemployed | 24 (7%) | 14 (8%) | 1 (2%) | 4 (7%) | 2 (11%) | 6 (5%) | 2 (4%) | | |
| Employee | 164 (46%) | 95 (54%) | 21 (46%) | 26 (43%) | 6 (33%) | 46 (38%) | 21 (46%) | | |
| Self-employed | 7 (2%) | 5 (3%) | 2 (4%) | 3 (5%) | 2 (11%) | 5 (4%) | 5 (11%) | | |
| Pensioner | 14 (4%) | 4 (2%) | 1 (2%) | 6 (10%) | 1 (6%) | 6 (5%) | 4 (9%) | | |
| Other | 16 (5%) | 5 (3%) | 3 (7%) | 2 (3%) | 3 (17%) | 6 (5%) | 2 (4%) | | |
| **People in the residency** | | | | | | | | 44.54, df = 55 | 0.842 |
| 1 | 64 (18%) | 26 (15%) | 4 (9%) | 11 (18%) | 4 (22%) | 28 (23%) | 9 (20%) | | |
| 2 | 136 (38%) | 64 (36%) | 24 (52%) | 25 (42%) | 5 (28%) | 41 (34%) | 18 (39%) | | |
| 3 | 68 (19%) | 35 (20%) | 6 (13%) | 10 (17%) | 4 (22%) | 24 (20%) | 7 (15%) | | |
| 4 | 55 (15%) | 36 (20%) | 5 (11%) | 7 (12%) | 2 (11%) | 14 (12%) | 6 (13%) | | |
| 5 or more | 32 (9%) | 15 (9%) | 7 /(15%) | 7 (12%) | 3 (17%) | 14 (12%) | 6 (13%) | | |
| **Children in the residency** | | | | | | | | 20.95, df = 20 | 0.400 |
| 0 | 259 (70%) | 112 (64%) | 37 (80%) | 43 (72%) | 12 (67%) | 93 (77%) | 33 (72%) | | |
| 1 | 44 (12%) | 24 (14%) | 3 (7%) | 6 (10%) | 2 (11%) | 14 (12%) | 5 (11%) | | |
| 2 | 49 (14%) | 34 (19%) | 3 (7%) | 7 (12%) | 3 (17%) | 11 (9%) | 6 (13%) | | |
| 3 | 8 (2%) | 4 (2%) | 2 (4%) | 3 (5%) | 1 (6%) | 2 (2%) | 1 (2%) | | |
| 4 or more | 4 (1%) | 2 (1%) | 1 (2%) | 1 (2%) | 0 (0%) | 1 (1%) | 1 (2%) | | |
| **Lifestyle and health related variables** | | | | | | | | | |

| | | | | | | | | | |
|---|---|---|---|---|---|---|---|---|---|
| **Diet type** | | | | | | | | 42.23, df = 25 | 0.017 |
| Omnivore | 245 (69%) | 131 (74%) | 28 (61%) | 45 (75%) | 8 (44%) | 78 (64%) | 31 (67%) | | |
| Flexitarian | 53 (15%) | 18 (10%) | 10 (22%) | 11 (18%) | 7 (39%) | 18 (15%) | 9 (20%) | | |
| Vegetarian | 14 (4%) | 5 (3%) | 4(9%) | 0 (0%) | 0 (0%) | 6 (5%) | 1 (2%) | | |
| Pescetarian | 20 (6%) | 13 (7%) | 3 (7%) | 2 (3%) | 1 (6%) | 8 (7%) | 3 (7%) | | |
| Vegan | 7 (2%) | 2 (1%) | 0 (0%) | 1 (2%) | 0 (0%) | 4 (3%) | 0 (0%) | | |
| Other | 16 (5%) | 7 (4%) | 1 (2%) | 0 (0%) | 2 (11%) | 7 (6%) | 2 (4%) | | |
| **BMI, mean (±SD)** | 25.03 (±5.57) | 25.00 (±5.27) | 24.43 (±4.81) | 26.35 (±5.97) | 23.85 (±4.44) | 25.44 (±6.45) | 26.57 (±6.54) | 309.21, df = 303 | 0.391* |
| **Physical Activity** | | | | | | | | 26.25, df = 20 | 0.158 |
| Bedbound | 1 (0%) | 1 (1%) | 0 (0%) | 0 (0%) | 0 (0%) | 0 (0%) | 0 (0%) | | |
| Very low activity | 18 (5%) | 10 (6%) | 1 (2%) | 3 (5%) | 1 (6%) | 4 (3%) | 1 (2%) | | |
| Low activity | 115 (32%) | 61 (35%) | 12 (26%) | 19 (32%) | 2 (11%) | 33 (27%) | 11 (24%) | | |
| Moderate | 164 (46%) | 79 (45%) | 25 (54%) | 30 (50%) | 6 (33%) | 55 (45%) | 23 (50%) | | |
| High activity | 57 (16%) | 25 (14%) | 8 (17%) | 8 (13%) | 9 (50%) | 29 (24%) | 11 (24%) | | |
| **Smoking** | | | | | | | | 9.58, df = 10 | 0.478 |
| Yes | 42 (12%) | 20 (11%) | 7 (15%) | 10 (17%) | 4 (22%) | 11 (9%) | 5 (11%) | | |
| No | 266 (75%) | 133 (76%) | 29 (63%) | 45 (75%) | 12 (67%) | 94 (78%) | 34 (74%) | | |
| Prior smoker | 47 (13%) | 23 (13%) | 10 (22%) | 5 (8%) | 2 (11%) | 16 (13%) | 7 (15%) | | |
| **Personality** | | | | | | | | | |
| Extrovert. Median (IQR) | 3 (2-4) | 3 (2-4) | 4 (2-4) | 3 (2-4) | 2.5 (2-3) | 3 (2-4) | 3 (2-4) | 36.20, df = 20 | 0.015 |
| Introvert. Median (IQR) | 3 (2-4) | 3 (2-4) | 2 (.25-3.75) | 3 (2-4) | 4 (3-4.75) | 3 (2-4) | 3 (2-4) | 35.29, df = 20 | 0.019 |
| **General health, Median (IQR)** | 3 (3-4) | 3 (3-4) | 3 (3-4) | 3 (3-4) | 3 (2.25-4) | 3 (3-4) | 3.5 (3-4) | 19.45, df = 20 | 0.493 |
| **Eating differently because Health Worries, now** | | | | | | | | 7.46, df = 5 | 0.189 |
| Yes | 69 (19%) | 142 (81%) | 8 (17%) | 15 (25%) | 4 (22%) | 26 (21%) | 13 (28%) | | |
| No | 283 (80%) | 32 (18%) | 37 (80%) | 45 (75%) | 13 (72%) | 94 (78%) | 31 (67%) | | |
| No comment | 3 (1%) | 2 (1%) | 1 (2%) | 0 (0%) | 1 (6%) | 1 (1%) | 2 (4%) | | |
| **Eating differently because Health Worries, prior** | | | | | | | | 8.76, df = 5 | 0.119 |
| Yes | 134 (38%) | 108 (61%) | 18 (39%) | 16 (27%) | 9 (50%) | 48 (40%) | 19 (41%) | | |
| No | 217 (61%) | 67 (38%) | 27 (59%) | 44 (73%) | 8 (44%) | 71 (59%) | 26 (57%) | | |
| No comment | 4 (1%) | 1 (1%) | 1 (2%) | 0 (0%) | 1 (6%) | 2 (2%) | 1 (2%) | | |
| **Alcohol consumption, Median (IQR)** | 2 (2-3) | 2 (2-3) | 3 (2-3) | 2 (2-3) | 2 (1.25-3) | 2 (2-3) | 2 (2-3) | 23.03, df = 20 | 0.287 |
| Never | 50 (14%) | 20 (11%) | 9 (20%) | 7 (12%) | 5 (28%) | 22 (18%) | 6 (13%) | | |
| Rarely | 148 (42%) | 73 (41%) | 12 (26%) | 27 (45%) | 7 (39%) | 54 (45%) | 20 (43%) | | |
| Sometimes | 135 (38%) | 73 (41%) | 20 (43%) | 19 (32%) | 4 (22%) | 40 (33%) | 17 (37%) | | |
| Often | 17 (5%) | 9 (5%) | 5 (11%) | 5 (8%) | 2 (11%) | 3 (2%) | 3 (7%) | | |
| Very often | 5 (1%) | 1 (1%) | 0 (0%) | 2 (3%) | 0 (0%) | 2 (2%) | 0 (0%) | | |
| **Addiction treatment, now** | | | | | | | | 214.54, df = 5 | <0.001 |

| | | | | | | | | | |
|---|---|---|---|---|---|---|---|---|---|
| Yes | 0 (0%) | 0 (0%) | 0 (0%) | 0 (0%) | 0 (0%) | 0 (0%) | 0 (0%) | | |
| No | 354 (100%) | 175 (99%) | 46 (100%) | 60 (100%) | 18 (100%) | 121 (100%) | 46 (100%) | | |
| No comment | 1 (0%) | 1 (1%) | 0 (0%) | 0 (0%) | 0 (0%) | 0 (0%) | 0 (0%) | | |
| **Addiction treatment, prior** | | | | | | | | 2.51, df = 5 | 0.776 |
| Yes | 4 (1%) | 2 (1%) | 0 (0%) | 0 (0%) | 0 (0%) | 2 (2%) | 0 (0%) | | |
| No | 350 (99%) | 173 (98%) | 46 (100%) | 60 (100%) | 18 (100%) | 119 (98%) | 46 (100%) | | |
| No comment | 1 (0%) | 1 (1%) | 0 (0%) | 0 (0%) | 0 (0%) | 0 (0%) | 0 (0%) | | |
| **Feeling stressed, Median (IQR)** | 3 (3-4) | 3 (3-4) | 3 (3-4) | 3 (3-4) | 3 (2.25-4) | 3 (2.75-4) | 3 (3-4) | 18.92, df = 20 | 0.527 |
| Never | 2 (1%) | 0 (0%) | 0 (0%) | 0 (0%) | 0 (0%) | 1 (1%) | 0 (0%) | | |
| Rarely | 79 (22%) | 40 (23%) | 11 (24%) | 11 (18%) | 5 (28%) | 28 (23%) | 10 (22%) | | |
| Sometimes | 151 (43%) | 88 (50%) | 17 (37%) | 25 (42%) | 5 (28%) | 45 (37%) | 20 (43%) | | |
| Often | 91 (26%) | 35 (20%) | 12 (26%) | 19 (32%) | 5 (28%) | 37 (31%) | 12 (26%) | | |
| Very often | 31 (9%) | 13 (7%) | 6 (13%) | 5 (8%) | 3 (17%) | 8 (7%) | 4 (9%) | | |
| **Stress treatment, now** | | | | | | | | 3.26, df = 5 | 0.661 |
| Yes | 13 (4%) | 6 (3%) | 1 (2%) | 0 (0%) | 3 (17%) | 5 (4%) | 2 (4% | | |
| No | 342 (96%) | 170 (97%) | 45 (98%) | 60 (100%) | 15 (83%) | 116 (96%) | 44 (96%) | | |
| No comment | 0 (0%) | 0 (0%) | 0 (0%) | 0 (0%) | 0 (0%) | 0 (0%) | 0 (0%) | | |
| **Stress treatment, prior** | | | | | | | | 5.16, df = 5 | 0.397 |
| Yes | 50 (14%) | 32 (18%) | 5 (11%) | 7 (12%) | 1 (6%) | 14 (12%) | 7 (15%) | | |
| No | 304 (86%) | 144 (82%) | 41 (89%) | 53 (88%) | 17 (94%) | 106 (87%) | 39 (85%) | | |
| No comment | 1 (0%) | 0 (0%) | 0 (0%) | 0 (0%) | 0 (0%) | 1 (1%) | 0 (0%) | | |
| **Feeling depressed, Median (IQR)** | 2 (2-3) | 2 (2-3) | 2 (2-3) | 3 (2-3) | 3 (3-4) | 3 (2-3) | 3 (2-3) | 23.50, df = 20 | 0.265 |
| Never | 48 (14%) | 24 (14%) | 8 (17%) | 10 (17%) | 1 (6%) | 19 (16%) | 1 (2%) | | |
| Rarely | 132 (37%) | 74 (42%) | 18 (39%) | 17 (28%) | 3 (17%) | 38 (31%) | 11 (24%) | | |
| Sometimes | 130 (37%) | 63 (36%) | 13 (28%) | 24 (40%) | 7 (39%) | 44 (36%) | 15 (33%) | | |
| Often | 35 (10%) | 12 (7%) | 4 (9%) | 7 (12%) | 6 (33%) | 15 (12%) | 8 (17%) | | |
| Very often | 9 (3%) | 2 (1%) | 2 (4%) | 1 (2%) | 0 (0%) | 4 (3%) | 0 (0%) | | |
| No comment | 1 (0%) | 1 (1%) | 1 (2%) | 1 (2%) | 1 (6%) | 1 (1%) | 1 (2%) | | |
| **Depression treatment, now** | | | | | | | | 19.24, df = 5 | 0.002 |
| Yes | 17 (5%) | 7 (4%) | 2 (4%) | 1 ((2%) | 4 (22%) | 5 (4%) | 2 (4%) | | |
| No | 335 (94%) | 167 (95%) | 43 (93%) | 58 (97%) | 12 (67%) | 114 (94%) | 42 (91%) | | |
| No comment | 3 (1%) | 2 (1%) | 1 (2%) | 1 (2%) | 2 (11%) | 2 (2%) | 2 (4%) | | |
| **Depression treatment, prior** | | | | | | | | 10.33, df = 5 | 0.066 |
| Yes | 60 (17%) | 30 (17%) | 10 (22%) | 10 (17%) | 7 (39%) | 17 (14%) | 11 (24%) | | |
| No | 292 (82%) | 144 (82%) | 35 (76%) | 49 (82%) | 9 (50%) | 102 (84%) | 33 (72%) | | |
| No comment | 3 (1%) | 2 (1%) | 1 (2%) | 1 (2%) | 2 (11%) | 2 (2%) | 2 (4%) | | |
| **Eating disorder treatment, now** | | | | | | | | 10.65, df = 5 | 0.059 |
| Yes | 5 (1%) | 0 (0%) | 1 (2%) | 2 (3%) | 1 (6%) | 2 (2%) | 1 (2%) | | |
| No | 348 (98%) | 175 (99%) | 45 (98%) | 57 (95%) | 16 (89%) | 118 (98%) | 44 (96%) | | |

| | | | | | | | | | |
|---|---|---|---|---|---|---|---|---|---|
| No comment | 2 (1%) | 1 (1%) | 0 (0%) | 1 (2%) | 1 (6%) | 1 (1%) | 1 (2%) | | |
| **Eating disorder treatment, prior** | | | | | | | | 8.70, df = 5 | 0.122 |
| Yes | 22 (6%) | 12 (7%) | 4 (9%) | 0 (0%) | 3 (17%) | 7 (6%) | 4 (9%) | | |
| No | 333 (94%) | 164 (93%) | 42 (91%) | 60 (100%) | 15 (83%) | 114 (94%) | 42 (91%) | | |
| No comment | 0 (0%) | 0 (0%) | 0 (0%) | 0 (0%) | 0 (0%) | 0 (0%) | 0 (0%) | | |
| **Diabetes treatment, Now** | | | | | | | | 26.03, df = 10 | 0.004 |
| Yes | 63 (18%) | 32 (18%) | 10 (22%) | 14 (23%) | 6 (33%) | 14 (12%) | 10 (22%) | | |
| No | 291 (82%) | 143 (81%) | 35 (76%) | 45 (75%) | 11 (61%) | 106 (88%) | 35 (76%) | | |
| No comment | 1 (0%) | 1 (1%) | 1 (2%) | 1 (2%) | 1 (6%) | 1 (1%) | 1 (2%) | | |
| **Diabetes treatment, Prior** | | | | | | | | 25.82, df = 5 | <0.001 |
| Yes | 38 (11%) | 15 (9%) | 7 (15%) | 11 (18%) | 5 (28%) | 8 (7%) | 7 (15%) | | |
| No | 316 (89%) | 160 (91%) | 39 (85%) | 49 (82%) | 13 (72%) | 113 (93%) | 39 (85%) | | |
| No comment | 1 (0%) | 1 (1%) | 0 (0%) | 0 (0%) | 0 (0%) | 0 (0%) | 0 (0%) | | |
| **Appetite and Eating Behaviour variables** | | | | | | | | | |
| **Disturbed Taste/odour** | | | | | | | | 3.07, df = 5 | 0.690 |
| Yes | 20 (6%) | 9 (5%) | 2 (4%) | 1 (2%) | 1 (6%) | 8 (7%) | 1 (2%) | | |
| No | 335 (94%) | 167 (95%) | 44 (96%) | 59 (98%) | 17 (94%) | 113 (93%) | 45 (98%) | | |
| **Disturbed food pleasure** | | | | | | | | 5.24, df = 5 | 0.387 |
| Yes | 53 (15%) | 20 (11%) | 8 (17%) | 8 (13%) | 4 (22%) | 19 (16%) | 5 (11%) | | |
| No | 302 (85%) | 156 (89%) | 38 (83%) | 52 (87%) | 14 (78%) | 102 (84%) | 41 (89%) | | |
| **General Appetite, Median (IQR)** | 3 (3-4) | 3 (2-4) | 3 (3-4) | 3 (2-4) | 3 (3-4) | 4 (3-4) | 3 (3-4) | 75.07, df = 20 | <0.001 |
| Very small | 1 (0%) | 0 (0%) | 0 (0%) | 0 (0%) | 1 (6%) | 0 (0%) | 0 (0%) | | |
| Small | 26 (7%) | 14 (8%) | 7 (15%) | 3 (5%9 | 3 (17%) | 5 (4%) | 3 (7%) | | |
| Regular | 184 (52%) | 102 (58%) | 20 (43%) | 38 (63%) | 6 (33%) | 53 (44%) | 24 (52%) | | |
| Large | 120 (34%) | 49 (28%) | 16 (35%) | 15 (25%) | 8 (44%) | 53 (44%) | 15 (33%) | | |
| Very large | 24 (7%) | 11 (6%) | 3 (7%) | 4 (7%) | 0 (0%) | 10 (8%) | 4 (9%) | | |
| **Food tastes…?, Median (IQR)** | 4 (4-5) | 4 (4-5) | 4 (4-5) | 4 (4-4.25) | 4 (3.25-4) | 4 (4-5) | 4 (4-5) | 16.93, df = 10 | 0.076 |
| Very bad | 0 (0%) | 0 (0%) | 0 (0%) | 0 (0%) | 0 (0%) | 0 (0%) | 0 (0%) | | |
| Bad | 0 (0%) | 0 (0%) | 0 (0%) | 0 (0%) | 0 (0%) | 0 (0%) | 0 (0%) | | |
| Okay | 31 (9%) | 11 (6%) | 7 (15%) | 8 (13%) | 5 (28%) | 12 (10%) | 6 (13%) | | |
| Good | 206 (58%) | 102 (58%) | 26 (57%) | 37 (62%) | 12 (67%) | 32 (26%) | 22 (48%) | | |
| Very good | 118 (33%) | 63 (36%) | 13 (28%) | 15 (25%) | 1 (6%) | 47 (39%) | 18 (39%) | | |
| **Difficulties with feeling hunger, Median (IQR)** | 2 (1-3) | 2 (1-3) | 2 (2-3) | 2 (2-2.25) | 2 (2-3) | 2 (1-2) | 2 (2-3) | 20.07, df = 20 | 0.453 |
| Agree very much | 5 (1%) | 3 (2%) | 2 (4%) | 1 (2%) | 1 (6%) | 2 (2%) | 1 (2%) | | |
| Agree | 35 (10%) | 17 (10%) | 9 (20%) | 6 (10%) | 8 (44%) | 8 (7%) | 4 (9%) | | |
| Neither nor | 51 (14%) | 28 (16%) | 8 (17%) | 8 (13%) | 4 (22%) | 16 13%) | 8 (17%) | | |
| Do not agree | 150 (42%) | 74 (42%) | 16 (35%) | 32 (53%) | 1 (6%) | 52 (43%) | 23 (50%) | | |
| Do not agree at all | 114 (32%) | 54 (31%) | 11 (24%) | 13 (22%) | 4 (22%) | 43 (36%) | 10 (22%) | | |

| | | | | | | | | | |
|---|---|---|---|---|---|---|---|---|---|
| **Difficulties with describing the feeling of hunger, Median (IQR)** | 2 (1-2) | 2 (1-2) | 2 (1.25-3) | 2 (2-3) | 2 (1.25-2.75) | 2 (1-2) | 2 (1-3) | 21.71, df = 20 | 0.357 |
| Agree very much | 2 (1%) | 1 (1%) | 0 (0%) | 0 (0%) | 0 (0%) | 1 (1%) | 0 (0%) | | |
| Agree | 31 (9%) | 15 (9%) | 9 (20%) | 5 (8%) | 0 (0%) | 9 (7%) | 5 (11%) | | |
| Neither nor | 44 (12%) | 13 (7%) | 10 (22%) | 12 (20%) | 5 (28%) | 19 (16%) | 8 (17%) | | |
| Do not agree | 161 (45%) | 79 (45%) | 15 (33%) | 33 (55%) | 8 (44%) | 51 (42%) | 18 (39%) | | |
| Do not agree at all | 117 (33%) | 68 (39%) | 12 (26%) | 10 (17%) | 5 (28%) | 41 (34%) | 15 (33%) | | |
| **Food Neophobia Scale, Mean (±SD)** | 41.12 (±6.63) | 41.39 (±6.49) | 43.15 (±5.07) | 41.23 (±6.68) | 33.11 (±8.65) | 40.39 (±6.93) | 40.11 (±8.06) | 50.56, df = 31 | 0.015* |
| **Dutch Eating Behavior Questionnaire** | | | | | | | | | |
| Restricted Eater, Mean (±SD) | 2.85 (±0.80) | 2.76 (±0.73) | 2.96 (±0.88) | 2.95 (±0.71) | 3.39 (±0.96) | 2.92 (±0.81) | 3.06 (±0.76) | 21.02, df = 20 | 0.396* |
| Emotional Eater, Mean (±SD) | 2.71 (±1.07) | 2.59 (±0.99) | 2.54 (±1.01) | 2.60 (±1.56) | 2.72 (±1.09) | 2.94 (±1.17) | 2.69 (±1.20) | 23.78, df = 24 | 0.474* |
| External Eater, Mean (±SD) | 3.27 (±0.66) | 3.27 (±0.60) | 3.17 (±0.53) | 3.16 (±0.69) | 2.90 (±0.95) | 3.41 (±0.72) | 3.31 (±0.76) | 22.32, df | 0.218* |
| **Food pleasure dimensions** | | | | | | | | | |
| Sensory-driven Pleasure, Mean (±SD) | 4.12 (±0.59) | 4.41 (±0.46) | 3.95 (±0.57) | 3.94 (±0.63) | 3.60 (±0.72) | 3.94 (±0.63) | 4.20 (±0.72) | 85.06, df = 14 | <0.001* |
| Exploratory-driven Pleasure, Mean (±SD) | 3.56 (±0.72) | 3.59 (±0.67) | 4.26 (±0.55) | 3.61 (±0.68) | 2.92 (±0.91) | 3.49 (±0.70) | 3.72 (±0.77) | 14.20, df = 16 | 0.584* |
| Contextual-driven Pleasure, Mean (±SD) | 3.53 (±0.79) | 3.52 (±0.76) | 3.57 (±0.77) | 4.32 (±0.55) | 3.09 (±0.87) | 3.42 (±0.76) | 3.92 (±0.77) | 13.06, df = 12 | 0.365* |
| Confirming-driven Pleasure, Mean (±SD) | 2.62 (±0.82) | 2.54 (±0.74) | 2.54 (±0.79) | 2.59 (±0.82) | 4.15 (±0.61) | 2.79 (±0.88) | 2.96 (±1.01) | 8.37, df = 12 | 0.756* |
| Internal-driven Pleasure, Mean (±SD) | 3.86 (±0.71) | 3.66 (±0.70) | 3.70 (±0.58) | 3.76 (±0.62) | 3.48 (±0.76) | 4.45 (±0.50) | 4.15 (±0.74) | 8.82, df = 10 | 0.550* |

*Kruskal-Wallis test

**Table S3**. Overview of ratings of each item (Median (Inter quartile range)) on a 5-point ordinal scale, where “1”=”Not important at all, “2”=”Not important”, “3”=”Neither important nor not important”, “4”=”Important” and “5”=”Extremely important”.

| Item | Median (Inter quartile range) |
|---|---|
| Food appearance | 4 (3-4) |
| Food odour | 4 (4-5) |
| Food taste | 5 (4-5) |
| Food texture | 4 (3-5) |
| Pleased senses | 4 (3-5) |
| Memory | 4 (3-4) |
| Novelty | 3 (3-4) |
| Surprise | 4 (3-4) |
| Variation | 4 (4-5) |
| Eating with others | 3 (3-4) |
| Physical surroundings | 3 (3-4) |
| Atmosphere | 4 (3-5) |
| Familiarity | 3 (2-3) |
| Eating alone | 2 (1.5-3) |
| Habit | 3 (2-3) |
| Physical sensation | 4 (3-4) |
| Mental sensation | 4 (3-4) |
| Need | 4 (4-5) |

**Table S4.** Multivariate Regression by Odd's Ratio on Sociodemographic variables for each of the five segments

| N=355 | Sensory Pleasure Seekers Odd's Ratio (CI) | p-value | Exploratory Pleasure Seekers Odd's Ratio (CI) | p-value | Contextual Pleasure Seekers Odd's Ratio (CI) | p-value | Confirming Pleasure Seekers Odd's Ratio (CI) | p-value | Internal Pleasure Seekers Odd's Ratio (CI) | p-value | Tie on first Odd's Ratio (CI) | p-value |
|---|---|---|---|---|---|---|---|---|---|---|---|---|
| **Sociodemographic variables** | | | | | | | | | | | | |
| **Gender** | | | | | | | | | | | | |
| **Female** | 1 | | 1 | | 1 | | 1 | | 1 | | 1 | |
| **Male** | 0.92 (0.54-1.55) | 0.750 | 1.30 (0.60-2.64) | 0.478 | 1.15 (0.66-1.97) | 0.615 | 1.13 (0.56-2.18) | 0.723 | 2.71 (0.97-7.17) | 0.047 | 1.70 (0.82-3.38) | 0.137 |
| **Age** | 1.02 (0.99-1.04) | 0.108 | 0.97 (0.93-1.00) | 0.062 | 0.99 (0.97-1.02) | 0.587 | 1.01 (0.98-1.04) | 0.403 | 1.04 (0.97-1.10) | 0.225 | 1.01 (0.98-1.04) | 0.479 |
| **Educational level** | | | | | | | | | | | | |
| **High School** | 1 | | 1 | | 1 | | 1 | | 1 | | | |
| **Primary School** | 3.63 (0.68-22.16) | 0.135 | 1.35 (1.06-10.85) | 0.802 | 0.32 (0.04-1.81) | 0.225 | NA | | NA | | NA | |
| **Vocational Education** | 7.00 (2.14-26.46) | 0.002 | 0.99 (0.13-4.91) | 0.992 | 0.20 (0.05-0.71) | 0.019 | 0.00 (0.00-0.00) | 0.991 | 0.39 (0.01-9.87) | 0.590 | 0.91 (0.11-5.02) | 0.920 |
| **Short Higher Education** | 5.12 (1.60-17.89) | 0.007 | 1.00 (0.13-5.06) | 0.998 | 0.41 (0.11-1.33) | 0.149 | 0.30 (0.01-2.07) | 0.299 | NA | | 1.58 (0.27-7.75) | 0.582 |
| **Med. Higher Education** | 4.51 (1.90-11.21) | <0.001 | 0.52 (0.10-2.00) | 0.378 | 0.21 (0.07-0.53) | 0.002 | 1.00 (0.27-3.51) | 1.000 | NA | | 0.50 (0.09-2.26) | 0.387 |
| **Long Higher Education** | 2.86 (1.30-6.45) | 0.009 | 1.31 (0.43-3.85) | 0.630 | 0.38 (0.16-0.86) | 0.022 | 1.82 (0.62-5.41) | 0.272 | 0.53 (0.04-6.17) | 0.601 | 2.35 (0.76-7.61) | 0.143 |
| **PhD** | 0.84 (0.11-4.35) | 0.846 | NA | | 0.76 (0.15-3.81) | 0.736 | 3.14 (0.47-18.38) | 0.210 | NA | | 1.09 (0.05-8.99) | 0.945 |
| **Socioeconomic Status** | | | | | | | | | | | | |
| **Employee** | 1 | | 1 | | 1 | | 1 | | 1 | | | |
| **Student** | 0.33 (0.17-0.65) | 0.001 | 1.24 (0.49-3.39) | 0.623 | 2.73 (1.35-5.68) | 0.006 | 0.96 (0.36-6.11) | 0.934 | 1.23 (0.02-12.28) | 0.783 | 0.72 (0.24-2.10) | 0.547 |
| **Unemployed** | 0.95 (0.29-3.39) | 0.939 | 0.59 (0.03-3.60) | 0.637 | 1.03 (0.22-3.75) | 0.963 | 1.15 (0.16-5.00) | 0.865 | NA | | 0.63 (0.03-3.74) | 0.676 |
| **Self-employed** | 0.58 (0.07-5.01) | 0.593 | 2.72 (0.13-24.40) | 0.410 | 10.08 (1.21-210.45) | 0.051 | 2.14 (0.10-18.21) | 0.524 | 19.36 (0.69-344.14) | 0.041 | 23.16 (2.72-489.94) | 0.009 |
| **Pensioner** | 0.58 (0.12-2.93) | 0.499 | NA | | 1.70 (0.29-8.62) | 0.526 | 1.79 (0.21-11.11) | 0.552 | 3.77 (0.07-193.56) | 0.484 | 2.82 (0.32-19.30) | 0.303 |
| **Other** | 0.48 (0.13-1.72) | 0.256 | 0.94 (0.05-5.89) | 0.955 | 2.63 (0.70-9.62) | 0.139 | 1.35 (0.19-6.11) | 0.721 | NA | | 0.79 (0.04-1.05) | 0.831 |
| **People in the residency** | | | | | | | | | | | | |

| | | | | | | | | | | | | |
|---|---|---|---|---|---|---|---|---|---|---|---|---|
| **1** | 0.59 (0.27-1.26) | 0.176 | 0.57 (0.15-1.78) | 0.364 | 2.52 (1.16-5.57) | 0.020 | 0.95 (0.33-2.47) | 0.912 | 7.41 (0.82-168.10) | 0.105 | 1.51 (0.28-3.98) | 0.413 |
| **2** | 1 | | 1 | | 1 | | 1 | | 1 | | | |
| **3** | 0.89 (0.42-1.88) | 0.763 | 0.40 (0.09-1.35) | 0.176 | 1.34 (0.61-2.91) | 0.464 | 0.52 (0.14-1.56) | 0.276 | NA | | 0.30 (0.05-1.17) | 0.126 |
| **4** | 1.77 (0.78-4.21) | 0.182 | 0.62 (0.13-2.13) | 0.479 | 0.64 (0.24-1.56) | 0.345 | 0.29 (0.04-1.11) | 0.112 | NA | | 0.34 (0.05-1.34) | 0.173 |
| **5 or more** | 0.73 (0.28-1.84) | 0.503 | 1.30 (0.37-3.98) | 0.661 | 0.79 (0.26-2.16) | 0.660 | 0.75 (0.16-2.57) | 0.668 | NA | | 0.28 (0.02-1.57) | 0.239 |
| **Children in the residency** | | | | | | | | | | | | |
| **0** | 1 | | 1 | | 1 | | 1 | | 1 | | | |
| **1** | 1.64 (0.72-3.83) | 0.242 | 0.55 (0.08-2.14) | 0.443 | 2.92 (1.16-8.45) | 0.032 | 0.54 (0.12-1.74) | 0.348 | NA | | 0.66 (0.14-2.17) | 0.533 |
| **2** | 3.97 (1.65-10.74) | 0.003 | 0.26 (0.01-1.44) | 0.210 | 2.45 (0.77-8.40) | 0.137 | 0.33 (0.05-1.25) | 0.154 | NA | | 0.38 (0.06-1.45) | 0.215 |
| **3** | 1.16 (0.21-6.48) | 0.859 | 1.46 (0.07-1.44) | 0.741 | 0.91 (0.04-7.23) | 0.935 | 1.00 (0.05-6.67) | 1.000 | NA | | NA | |
| **4 or more** | 2.36 (0.22-51.79) | 0.859 | 3.79 (0.17-44.13) | 0.296 | NA | | 2.28 (0.10-25.10) | 0.512 | NA | | 2.90 (0.13-32.07) | 0.397 |
| **Lifestyle and health variables** | | | | | | | | | | | | |
| **Diet type** | | | | | | | | | | | | |
| Omnivore | 1 | | 1 | | 1 | | 1 | | 1 | | 1 | |
| Flexitarian | 0.51 (0.23-1.08) | 0.082 | 1.61 (0.53-4.39) | 0.374 | 2.06 (0.77-5.15) | 0.130 | 5.50 (0.62-2.32) | 0.101 | 1.03 (0.46-2.23) | 0.941 | 1.34 (0.41-3.75) | 0.598 |
| Vegetarian | 0.50 (0.12-1.75) | 0.290 | 4.31 (1.00-16.66) | 0.037 | NA | | NA | | 1.13 (0.28-3.95) | 0.856 | 0.82 (0.04-4.75) | 0.851 |
| Pescetarian | 1.46 (0.50-4.58) | 0.496 | 1.06 (0.15-4.51) | 0.943 | 0.51 (0.03-2.88) | 0.524 | NA | | 1.13 (0.36-3.31) | 0.824 | 1.21 (0.18-5.05) | 0.819 |
| Vegan | 0.33 (0.05-1.59) | 0.194 | NA | | 1.15 (0.06-7.31) | 0.902 | NA | | 2.64 (0.56-13.87) | 0.217 | NA | |
| Other | 1.51 (0.38-7.43) | 0.571 | 1.23 (0.06-7.67) | 0.854 | 0.80 (0.04-4.81) | 0.838 | NA | | 0.60 (0.08-2.47) | 0.494 | 0.91 (0.05-2.02) | 0.932 |
| **BMI** | **0.99 (0.94-1.04)** | **0.774** | 0.99 (0.90-1.06) | 0.733 | 0.99 (0.91-1.06) | 0.768 | 0.96 (0.73-1.13) | 0.735 | 1.04 (0.99-1.10) | 0.100 | 1.01 (0.97-1.10) | 0.213 |
| **Physical Activity** | | | | | | | | | | | | |
| Bedbound | NA | | NA | | NA | | NA | | NA | | NA | |
| Very low activity | 1.81 (0.51-7.31) | 0.371 | 0.61 (0.03-3.62) | 0.646 | 0.49 (0.03-2.84) | 0.511 | NA | | 0.73 (0.15-2.72) | 0.661 | 0.60 (0.03-3.54) | 0.644 |
| Low activity | 1.17 | 0.590 | 0.79 | 0.591 | 0.87 | 0.740 | 0.40 | 0.448 | 0.85 | 0.599 | 0.68 | 0.421 |

| | | | | | | | | | | | | |
|---|---|---|---|---|---|---|---|---|---|---|---|---|
| | (0.65-2.11) | | (0.32-1.86) | | (0.38-1.94) | | (0.02-3.42) | | (0.45-1.57) | | (0.26-1.68) | |
| Moderate | 1 | | 1 | | 1 | | 1 | | 1 | | 1 | |
| High activity | 1.25 (0.54-2.96) | 0.605 | 0.48 (0.07-1.90) | 0.360 | 0.13 (0.01-1.37) | 0.059 | 0.49 (0.02-5.92) | 0.619 | 1.74 (0.73-4.13) | 0.207 | 0.64 (0.13-2.23) | 0.517 |
| **Smoking** | | | | | | | | | | | | |
| No | 1 | | 1 | | 1 | | 1 | | 1 | | 1 | |
| Yes | 1.35 (0.58-3.19) | 0.485 | 0.65 (0.10-2.45) | 0.576 | 2.08 (0.70-5.54) | 0.161 | 3.34 (0.15-32.23) | 0.326 | 0.50 (0.17-1.24) | 0.158 | 1.02 (0.23-3.28) | 0.981 |
| Prior smoker | 1.17 (0.50-2.76) | 0.723 | 3.41 (1.05-10.48) | 0.033 | 0.65 (0.14-2.21) | 0.532 | 1.72 (0.07-16.90) | 0.668 | 0.54 (0.19-1.34) | 0.201 | 1.11 (0.28-3.49) | 0.873 |
| **Personality** | | | | | | | | | | | | |
| Extrovert | 1.20 (1.96-1.52) | 0.108 | 1.18 (0.84-1.67) | 0.343 | 1.37 (0.98-1.93) | 0.069 | 0.35 (0.10-1.11) | 0.058 | 0.78 (0.61-0.99) | 0.048 | 1.13 (0.80-1.61) | 0.500 |
| Introvert | 0.85 (0.67-1.07) | 0.159 | 0.69 (0.48-0.97) | 0.033 | 0.83 (0.59-1.15) | 0.256 | 6.83 (1.79-42.79) | 0.016 | 1.36 (1.06-1.76) | 0.016 | 0.96 (0.68-1.38) | 0.838 |
| **General health** | **0.92 (0.69-1.23)** | 0.578 | 1.14 (0.73-1.81) | 0.564 | 1.12 (0.74-1.70) | 0.583 | 1.227 (0.50-3.39) | 0.619 | 1.09 (0.80-1.48) | 0.578 | 1.21 (0.78-1.90) | 0.401 |
| **Eating differently because of Health Worries. now** | | | | | | | | | | | | |
| No | 1 | | 1 | | 1 | | 1 | | 1 | | 1 | |
| Yes | 1.01 (0.50-2.04) | 0.975 | 1.25 (0.39-3.42) | 0.688 | 1.25 (0.45-3.08) | 0.648 | NA | | 1.32 (0.64-2.69) | 0.450 | 1.72 (0.61-4.44) | 0.279 |
| **Eating differently because of Health Worries. prior** | | | | | | | | | | | | |
| No | 1 | | 1 | | 1 | | 1 | | 1 | | 1 | |
| Yes | 1.17 (0.68-2.04) | 0.571 | 1.40 (0.61-3.20) | 0.419 | 0.45 (0.17-5.85) | 0.079 | 2.83 (0.30-27.38) | 0.334 | 1.11 (0.62-1.96) | 0.720 | 1.32 (0.56-3.05) | 0.516 |
| **Alcohol consumption** | | | | | | | | | | | | |
| Never | 0.63 (0.25-1.50) | 0.306 | 1.79 (0.49-5.89) | 0.348 | 0.74 (0.20-2.29) | 0.628 | 1.39 (0.06-16.45) | 0.799 | 1.33 (0.56-3.13) | 0.510 | 0.80 (0.17-2.77) | 0.745 |
| Rarely | 1 | | 1 | | 1 | | 1 | | 1 | | 1 | |
| Sometimes | 1.71 (0.96-3.09) | 0.071 | 1.83 (0.76-4.62) | 0.183 | 0.52 (0.21-1.23) | 0.146 | 0.49 (0.023-5.39) | 0.568 | 0.56 (0.30-1.04) | 0.068 | 0.84 (0.34-2.03) | 0.700 |
| Often | 2.28 (0.42-17.23) | 0.359 | NA | | 2.25 (0.29-12.87) | 0.380 | 6.89 (0.24-104.53) | 0.176 | NA | | 1.32 (0.07-9.25) | 0.809 |
| Very often | 0.36 (0.02-4.08) | 0.422 | NA | | NA | | NA | | 4.19 (037-96.63) | 0.263 | NA | |
| **Addiction treatment. now** | | | | | | | | | | | | |
| No | 1 | | 1 | | 1 | | 1 | | 1 | | 1 | |
| Yes | NA | | NA | | NA | | NA | | NA | | NA | |
| **Addiction treatment. prior** | | | | | | | | | | | | |

| | | | | | | | | | | | | |
|---|---|---|---|---|---|---|---|---|---|---|---|---|
| No | 1 | | 1 | | 1 | | 1 | | 1 | | 1 | |
| Yes | 1.61 (0.06-42.74) | 0.743 | NA | | NA | | NA | | 2.40 (0.09-64.34) | 0.548 | NA | |
| **Feeling stressed** | | | | | | | | | | | | |
| Never | NA | | NA | | NA | | NA | | NA | | NA | |
| Rarely | 0.64 (0.33-1.25) | 0.189 | 1.44 (0.53-3.81) | 0.461 | 0.50 (0.17-1.34) | 0.190 | 0.51 (0.02-5.80) | 0.595 | 1.10 (0.53-2.24) | 0.799 | 0.27 (0.06-0.87) | 0.047 |
| Sometimes | 1 | | 1 | | 1 | | 1 | | 1 | | 1 | |
| Often | 0.40 (0.20-0.79) | 0.009 | 1.06 (0.36-2.93) | 0.909 | 0.76 (0.27-.96) | 0.582 | 1.87 (0.08-27.58) | 0.645 | 1.92 (0.97-4.83) | 0.063 | 0.66 (0.22-1.76) | 0.420 |
| Very often | 0.80 (0.27-2.43) | 0.681 | 1.13 (0.16-4.94) | 0.884 | 1.32 (0.28-4.80) | 0.698 | 9.02 (0.33-186.45) | 0.133 | 0.78 (0.20-2.49) | 0.695 | 1.34 (0.28-4.91) | 0.680 |
| **Stress treatment. now** | | | | | | | | | | | | |
| No | 1 | | 1 | | 1 | | 1 | | 1 | | 1 | |
| Yes | 1.58 (0.37-7.91) | 0.543 | NA | | NA | | NA | | 1.09 (0.21-4.61) | 0.906 | NA | |
| **Stress treatment. prior** | | | | | | | | | | | | |
| No | 1 | | 1 | | 1 | | 1 | | 1 | | 1 | |
| Yes | 2.73 (1.18-6.89) | 0.024 | 0.56 (0.09-2.10) | 0.454 | 0.38 (0.06-1.42) | 0.214 | NA | | 0.51 (0.19-1.21) | 0.146 | 0.73 (0.16-2.36) | 0.630 |
| **Feeling depressed** | | | | | | | | | | | | |
| Never | 0.81 (0.37-1.81) | 0.611 | 2.45 (0.85-6.91) | 0.090 | 1.11 (0.33-3.30 | 0.857 | NA | | 1.63 (0.70-3.73) | 0.248 | 3.11 (0.98-9.92) | 0.050 |
| Rarely | 1 | | 1 | | 1 | | 1 | | 1 | | 1 | |
| Sometimes | 0.99 (0.54-1.84) | 0.984 | 0.59 (0.19-1.65) | 0.326 | 1.06 (0.42-2.63) | 0.900 | 0.59 (0.03-6.75) | 0.681 | 1.32 (0.68-2.56) | 0.405 | 1.29 (0.42-3.95) | 0.652 |
| Often | 0.53 (0.20-1.32) | 0.180 | 1.24 (0.31-4.18) | 0.737 | 1.49 (0.38-4.93) | 0.530 | 8.40 (0.82-96.55) | 0.064 | 2.11 (0.82-5.38) | 0.116 | 4.47 (1.28-15.43) | 0.017 |
| Very often | 0.29 (0.01-2.42) | 0.296 | NA | | 2.37 (0.11-20.59) | 0.473 | NA | | 2.57 (0.30-22.50) | 0.358 | NA | |
| **Depression treatment. now** | | | | | | | | | | | | |
| No | 1 | | 1 | | 1 | | 1 | | 1 | | 1 | |
| Yes | 1.43 (0.40-5.78) | 0.587 | 1.82 (0.26-7.95) | 0.468 | 0.69 (0.04-3.87) | 0.726 | NA | | 0.48 (0.07-1.98) | 0.360 | 0.88 (0.05-4.99) | 0.904 |
| **Depression treatment. prior** | | | | | | | | | | | | |
| No | 1 | | 1 | | 1 | | 1 | | 1 | | 1 | |
| Yes | 1.04 (0.51-2.10) | 0.922 | 1.91 (0.70-4.81) | 0.182 | 1.11 (0.39-2.79) | 0.830 | 5.02 (0.52-48.20) | 0.136 | 0.64 (0.28-1.37) | 0.270 | 1.48 (0.50-3.82) | 0.441 |
| **Eating disorder treatment. now** | | | | | | | | | | | | |

| | | | | | | | | | | | | |
|---|---|---|---|---|---|---|---|---|---|---|---|---|
| No | 1 | | 1 | | 1 | | 1 | | 1 | | 1 | |
| Yes | NA | | NA | | NA | | NA | | NA | | NA | |
| **Eating disorder treatment. prior** | | | | | | | | | | | | |
| No | 1 | | 1 | | 1 | | 1 | | 1 | | 1 | |
| Yes | 1.57 (0.50-5.42) | 0.448 | 1.26 (0.18-5.22) | 0.780 | NA | | 17.75 (0.65-487.43) | 0.051 | 1.60 (0.62-5.07) | 0.416 | 4.29 (1.06-15.10) | 0.028 |
| **Diabetes treatment. now** | | | | | | | | | | | | |
| No | 1 | | 1 | | 1 | | 1 | | 1 | | 1 | |
| Yes | 1.55 (0.66-3.77) | 0.320 | 5.68 (1.51-21.82) | 0.010 | 0.74 (0.19-2.37) | 0.630 | NA | | 0.27 (0.08-0.73) | 0.016 | 0.88 (0.23-3.02) | 0.891 |
| **Diabetes treatment. prior** | | | | | | | | | | | | |
| No | 1 | | 1 | | 1 | | 1 | | 1 | | 1 | |
| Yes | 1.03 (0.38-2.87) | 0.950 | 6.65 (1.70-25.74) | 0.005 | 1.13 (0.24-3.95) | 0.859 | NA | | 0.32 (0.07-1.03) | 0.083 | 1.33 (0.28-1.74) | 0.684 |
| **Appetite and eating behaviour variables** | | | | | | | | | | | | |
| **Disturbed Taste/olfactory sense** | | | | | | | | | | | | |
| **No** | 1 | | 1 | | 1 | | 1 | | 1 | | 1 | |
| **Yes** | 1.58 (0.45-6.29) | 0.482 | 0.76 (0.04-4.44) | 0.801 | NA | | NA | | 0.76 (0.16-2.74) | 0.694 | NA | |
| **Disturbed Food Pleasure** | | | | | | | | | | | | |
| **No** | 1 | | 1 | | 1 | | 1 | | 1 | | | |
| **Yes** | 0.78 (0.33-1.82) | 0.561 | 1.62 (0.43-4.95) | 0.426 | NA | | 3.56 (0.16-41.18) | 0.318 | 1.29 (0.53-3.03) | 0.559 | 0.58 (0.10-10.44) | 0.542 |
| **General appetite** | | | | | | | | | | | | |
| Very small | NA | | NA | | NA | | NA | | NA | | NA | |
| Small | 1.09 (0.35-3.61) | 0.881 | 0.87 (0.12-3.81) | 0.864 | 0.95 (0.14-4.13) | 0.955 | NA | | 0.77 (0.16-2.76) | 0.712 | 0.80 (0.04-4.93) | 0.842 |
| Regular | 1 | | 1 | | 1 | | 1 | | 1 | | 1 | |
| Large | 0.55 (0.30-1.01) | 0.055 | 0.96 (0.39-2.30) | 0.926 | 0.63 (0.24-1.53) | 0.319 | 10.96 (1.32-251.10) | 0.053 | 2.14 (1.14-4.07) | 0.019 | 1.44 (0.55-3.71) | 0.449 |
| Very large | 0.52 (0.19-1.39) | 0.195 | 0.82 (0.12-3.39) | 0.810 | 0.97 (0.21-3.30) | 0.963 | NA | | 3.23 (1.19-8.89) | 0.021 | 2.41 (0.61-8.04) | 0.171 |
| **Food tastes…?** | | | | | | | | | | | | |
| Very bad | NA | | NA | | NA | | NA | | NA | | NA | |
| Bad | NA | | NA | | NA | | NA | | NA | | NA | |
| Okay | 0.63 (0.18-2.10) | 0.456 | 1.52 (0.22-6.67) | 0.617 | 1.05 (0.15-4.40) | 0.955 | 2.58 (0.12-22.32) | 0.440 | 1.20 (0.31-4.07) | 0.776 | 1.83 (0.16-8.01) | 0.468 |
| Good | 1 | | 1 | | 1 | | 1 | | 1 | | 1 | |

| | | | | | | | | | | | | |
|---|---|---|---|---|---|---|---|---|---|---|---|---|
| Very good | 1.07 (0.62-1.87) | 0.806 | 0.86 (0.35-1.99) | 0.723 | 0.70 (0.29-1.58) | 0.404 | NA | | 1.55 (0.87-2.76) | 0.136 | 1.64 (0.70-3.84) | 0.248 |
| **Difficulties with feeling hunger** | 1.06 (0.81-1.37) | 0.682 | 1.33 (0.91-1.91) | 0.133 | 1.21 (0.85-1.71) | 0.277 | 0.68 (0.17-1.74) | 0.495 | 0.77 (0.57-1.02) | 0.073 | 1.03 (0.68-1.50) | 0.892 |
| **Difficulties with describing the feeling of hunger** | 0.77 (0.57-1.03) | 0.088 | 1.27 (0.84-1.88) | 0.243 | 1.38 (0.93-2.02) | 0.099 | 1.24 (0.47-2.81) | 0.622 | 0.97 (0.71-1.31) | 0.851 | 1.16 (0.75-1.76) | 0.485 |
| **Food Neophobia Scale** | 1.02 (0.97-10.7) | 0.391 | 1.05 (0.97-1.14) | 0.217 | 1.05 (0.98-1.13) | 0.205 | 0.82 (0.69-0.94) | 0.007 | 0.97 (0.92-1.02) | 0.188 | 1.02 (0.95-1.10) | 0.672 |
| **Dutch Eating Behaviour Questionnaire** | | | | | | | | | | | | |
| Restricted Eater | 0.87 (0.62-1.22) | 0.429 | 1.06 (0.64-1.75) | 0.832 | 1.01 (0.61-1.64) | 0.981 | 4.44 (1.37-17.94) | 0.019 | 1.04 (0.73-1.49) | 0.821 | 1.21 (0.72-2.06) | 0.471 |
| Emotional Eater | 0.81 (0.62-1.04) | 0.101 | 0.88 (0.59-1.31) | 0.538 | 1.00 (0.68-1.46) | 0.999 | 1.42 (0.52-4.07) | 0.488 | 1.37 (1.04-1.81) | 0.025 | 1.13 (0.76-1.69) | 0.534 |
| External Eater | 0.80 (0.53-1.21) | 0.296 | 0.72 (0.37-1.39) | 0.335 | 1.11 (0.61-2.01) | 0.729 | 1.40 (0.35-6.03) | 0.638 | 1.64 (1.06-2.59) | 0.030 | 1.55 (0.82-2.93) | 0.176 |
| **Food pleasure dimensions** | | | | | | | | | | | | |
| Sensory Pleasure | 23.60 (10.77-57.56) | <0.001 | 0.42 (0.20-0.87) | 0.022 | 0.46 (0.22-0.93) | 0.031 | 0.14 (0.02-0.84) | 0.039 | 0.29 (0.16-0.50) | <0.001 | 1.34 (0.71-3.45) | 0.294 |
| Exploratory Pleasure | 1.22 (0.82-1.84) | 0.333 | 15.94 (5.82-52.39) | <0.001 | 1.39 (0.77-2.63) | 0.290 | 0.29 (0.08-0.93) | 0.034 | 0.50 (0.32-0.77) | 0.002 | 1.97 (1.00-4.15) | 0.062 |
| Contextual Pleasure | 1.24 (0.88-1.76) | 0.229 | 0.92 (0.56-1.52) | 0.728 | 20.27 (7.86-63.33) | <0.001 | 0.55 (0.17-1.78) | 0.312 | 0.56 (0.38-0.81) | 0.003 | 2.34 (1.29-4.52) | 0.008 |
| Confirming Pleasure | 0.80 (0.56-1.14) | 0.216 | 0.79 (0.45-1.36) | 0.408 | 0.76 (0.45-1.26) | 0.299 | 67.33 (8.21-2191.70) | 0.002 | 1.52 (1.05-2.22) | 0.027 | 1.49 (0.88-2.52) | 0.136 |
| Internal Pleasure | 0.41 (0.26-0.62) | <0.001 | 0.52 (0.29-0.92) | 0.025 | 0.61 (0.35-1.04) | 0.069 | 0.68 (0.18-2.76) | 0.570 | 27.63 (12.55-69.00) | <0.001 | 1.81 (0.98-3.50) | 0.065 |
